# Experimental evaluation of the influence of combined particle size pretreatment and Fe_3_O_4_ additive on fuel yields of *Arachis Hypogea* shells

**DOI:** 10.1177/0734242X221122560

**Published:** 2022-09-20

**Authors:** Kehinde O Olatunji, Daniel M Madyira, Noor A Ahmed, Oyetola Ogunkunle

**Affiliations:** Department of Mechanical Engineering Science, Faculty of Engineering and the Built Environment, University of Johannesburg, Johannesburg, South Africa

**Keywords:** Anaerobic digestion, lignocellulose, pretreatment, biogas, methane

## Abstract

A smart energy recovery process can achieve maximum energy recovery from organic wastes. Pretreatment of feedstock is essential to biogas and methane yields during the anaerobic digestion process. This work combined particle size reduction with Fe_3_O_4_ nanoparticles to investigate their influence on biogas and methane yields from anaerobic digestion of *Arachis hypogea* shells. Twenty milligrams per litre of Fe_3_O_4_ nanoparticles was implemented with 2, 4, 6 and 8 mm particle sizes and a single treatment of Fe_3_O_4_ for 35 days. The treatments were compared with each other and were discovered to significantly (*p* < 0.05) enhance biogas yield by 37.40%, 50.10%, 54.40%, 51.40% and 35.50% compared with control, respectively. Specific biogas yield recorded was 966.2, 1406, 1552.7, 1317.4, 766.2 and 413 mL g^−1^ volatile solid. This study showed the combination of Fe_3_O_4_ with 6 mm particle size of *Arachis hypogea* shells produced the optimum biogas and methane yields. The addition of Fe3O4 to particle sizes below 6 mm resulted in over-accumulation of volatile fatty acids and lowered the gas yield. This can be applied on an industrial scale.

## Introduction

Economic development globally has resulted in an extensive consumption of fossil fuels, leading to higher carbon dioxide emissions into the environment. The higher carbon dioxide release effects on the infrared rays have resulted in greenhouse challenges (Damyanova and Beschkov, 2020). These challenges have necessitated several research efforts to produce sustainable and green energy to minimize the environmental problem due to the consumption of fossil fuels. Renewable biofuels from organic wastes have been discovered as energy sources that can substitute fossil fuels and lower greenhouse gas emissions. Biogas produced from anaerobic digestion of various organic wastes is one biofuel that can potentially replace these fuels with high carbon content (Ogunkunle et al., 2018). An anaerobic digestion’s main benefits include combined energy and environmental effect. Digestate from anaerobic digestion can serve as a source of organic manure in agriculture (Maroušek et al., 2022). The process remains an effective and valuable technology for converting biodegradable wastes into energy. Although the anaerobic digestion process is an effective waste-to-energy approach, it is time-consuming due to the nature of the feedstocks (Olatunji et al., 2022c). The microbial present in the process may prefer particular feedstock composition over others (Yue et al., 2010). Due to the recalcitrance nature of some feedstock, there are losses of carbon content that are expected to be converted to biogas (Saini et al., 2015).

The principal feedstocks for anaerobic digestion are waste from agricultural activities (crop residues, animal wastes, etc.), activated sludge, waste from the food industry, landfill gas, stillage from ethanol production, etc. (Cesaro and Belgiorno, 2015; Ogunkunle et al., 2019). Biomass is the fourth largest global primary energy source contributing about 14%, and it can be as higher as 35% in developing countries (Khanal et al., 2019). Agricultural biomass has been reported to have a higher potential for energy generation (Surendra et al., 2014). Reports have shown that countries such as China, Germany, Brazil, etc., utilize anaerobic digestion technology to produce energy from organic wastes. In contrast, many African countries still depend on traditional means of biomass usage, thereby hindering the capacity of energy that can be recovered from the vast amount of organic wastes available (Tagne et al., 2021). Using these residues as feedstock for energy production will lower the cost of waste management and energy cost (Škapa and Vochozka, 2019).

The majority of the residues from agricultural activities are lignocellulosic, and they are the most available renewable feedstock on earth. The principal components of lignocellulose are cellulose, hemicellulose and lignin, which are firmly attached (Kumar and Sharma, 2017). Anaerobic digestion of lignocellulose materials involves biological and chemical processes; this includes breaking down bigger organic polymers that form the biomass into smaller molecules with the catalytic activities of microbes and chemicals. In biogas and methane production, four stages of anaerobic digestion are required: hydrolysis, acidogenesis, acetogenesis and methanogenesis (Raja and Wazir, 2017). It is vital to note that some lignocellulose feedstock is not easy to degrade and accessible to bacteria during the hydrolysis stage because of their complex structures (Lin et al., 2010).

The hydrolysis stage has been recorded to hinder the anaerobic digestion process of lignocellulose due to their recalcitrant structure (Taherzadeh and Karimi, 2008). The research has shown that the methanogenesis stage can also be a rate-limiting step dependent on the proportion of hydrolytic to methanogenic microbes (Luo et al., 2012). Because of the degree of importance of the hydrolysis stage in the kinetics of anaerobic digestion, special attention has been given to techniques that can expedite the hydrolysis stage during anaerobic digestion. Different pretreatment methods are being investigated and applied to influence the hydrolysis stage, particularly feedstock with high resistance to enzymatic attack (Kumar and Sharma, 2017). Pretreatment techniques are mainly involved in efficient dissociation of the complexly interlinked portions and improving the availability of the different components. The main hurdle in the pretreatment process is eliminating sturdy and rugged lignin content that hinders their solubilization, and restraint hydrolysis of cellulose and hemicellulose. But the release of inhibitory products during pretreatment and feedstock particle size also limits the digestion of lignocellulose feedstock (Kumar and Sharma, 2017). The most popular pretreatment techniques include biological, chemical, thermal, mechanical/physical and combined pretreatment techniques. It has been reported that pretreating all feedstock with a single approach is not realistic because various lignocellulose materials were said to have different reactions to the pretreatment method or are uneconomically viable. The appropriate method can be selected based on the available feedstocks and techniques, but the process must be adequate, economical and suits the expected yields (Olatunji et al., 2021). The choice of pretreatment techniques devolves mainly on feedstock, application and cost.

The interdisciplinary investigation in nano-science and technology has recently received more attention globally. It has been discovered that nanomaterials can revolutionize the structural component of materials and products, and enhance their availability. Investigations have shown that some nanoparticles can absorb and/or react with cell membranes and rupture them. Nanoparticles interfered with the substrate microbial homeostasis attached and improve the microbial species robustness and community diversity (Zhou et al., 2021). Nanoparticles of metal origin could aggregate and create a serious decrease in the size of the feedstocks (Zhou et al., 2019b). Nanomaterials can enhance the efficiency of blocked enzymes since they supply sufficient surface area for the enzyme affixation, which increases enzyme attachment per unit mass of particles (Grewal et al., 2017). It was noticed that nanoparticles bonded with acetic acid were produced during the anaerobic digestion process mainly by the van der Waals force (Zhou et al., 2019a). The addition of nanoparticles can improve the nutritional content of fermentation residues which can be used for fertilization purposes (Maroušek and Gavurová, 2022). The commercial application of nanoparticles in consumer and industrial production processes has raised anxieties concerning their possible effects on the environment. Consequently, the influence of different nanoparticles (Fe_2_O_3_, MgO, Ag, nano zero-valent iron, etc,) on the anaerobic digestion of lignocellulose feedstock needs special attention.

The recent knowledge about direct interspecies electron transfer (DIET) in methanogenic environments has been reported as a result of electric syntrophy between exo-electrogenic *Geobacter* species and methanogenic bacteria (Rotaru et al., 2014; Shrestha et al., 2014). The microbial community composition showed a dominance of *Methanosaeta concilii* and *Geobacter* species in the aggregates considered, and the *Methanosaeta* species are mainly acetoclastic methanogen (Garcia et al., 2000). *Geobacter* species are capable of degrading simple organic acids and extracellularly exchanging electrons with their syntrophic partners when there are no conductive solids or insoluble electron acceptors (Summers et al., 2010). The application of iron nanoparticles like hematite and magnetite has shown their ability to act as heterogeneous catalysis in different application areas, including anaerobic digestion. They can facilitate extracellular electron transport by iron-reducing microorganisms such as *Geobacter* species (Kato et al., 2012). The addition of iron nanoparticle during anaerobic digestion was reported to significantly lower the lag time and enhance biogas and methane yields due to a DIET-based syntropy (Kato et al., 2012). Iron nanoparticles-supplemented methanogenic feedstocks have been reported to show complex aggregate arrangement due to extensive colonization of microorganisms. This improves the process’s lag time and methane yield (Baek et al., 2017). Table 1 shows some of the applications of nanoparticles in the biogas production process. It can be inferred from the table that various nanomaterials have different influences on individual feedstock for optimum biogas and methane yields. Other effects of the nanoparticles during pretreatment and biogas yields necessitated the establishment of new guidelines for the application of different nanoparticles to improve the anaerobic digestion and minimize inhibitory products. As for the use of nanoparticles for lignocellulose pretreatment, it can be seen that there is limited literature. With the importance of lignocellulose feedstocks in biofuel production, there is an urgent need to investigate the effect of this exceptional pretreatment method that has been researched and reported to be convincing in sludge pretreatment.

**Table 1.** Effect of nanoparticles pretreatment on biogas and methane yields.

S/N	Feedstock	Nano-treatment	Effect	Reference
1	Sludge from UASB reactor	CeO_2_	Increase in biogas yield	Duc (2013)
2	Fresh raw manure	Co	Increase in biogas and methane yields	Abdelsalam et al. (2016)
3	WAS	ZnO	No effect	Mu et al. (2012)
4	WAS	Fe_2_O_3_	Increase in methane yield	Wang et al. (2016)
5	WAS	Ag	No effect	Doolette et al. (2013)
6	WAS	TiO_2_	No effect	Cheng et al. (2014)
7	AGS	CuO	Decrease in methane yield	Otero-González et al. (2014)
8	Sludge from UASB reactor	ZnO	Decrease in biogas yield	Duc (2013)
9	Fresh raw manure	Fe_3_O_4_	Increase in biogas and methane yields	Abdelsalam et al. (2016)
10	Cattle slurry	Ni	Increase in biogas and methane yields	Abdelsalam et al. (2016)

*Arachis Hpogea* shell is one of the abundant feedstocks that have been investigated to have an excellent potential for biogas and methane generation (Jekayinfa et al., 2020). The study shows that different particle sizes of *Arachis hypogea* significantly influence the biogas yield, and the expected yield determines the choice of particle size (Olatunji et al., 2022b). Compared with the conventional single pretreatment method, a combination of two or more pretreatment methods is advantageous in minimizing operational process stages apart from reducing the retention period and release of undesirable inhibitors (Kumar and Sharma, 2017). Reducing the retention period is a significant advantage that can be regarded as crucial economic savings on digester volume size and substrate handling (Maroušek, 2014). Particle size reduction is an essential pretreatment method during the anaerobic digestion of most lignocellulose materials due to their sizes. Most of these materials required size reduction before anaerobic digestion or pretreatment with other techniques. Hence, it is vital to establish the appropriate particle size for the optimum biogas and methane yields for Fe_3_O_4_ nanoparticle additive. A proper selection of the process parameters that can be replicated on an industrial level would enable us to determine the astuteness dynamics of the anaerobic digestion process by straightforward and simple optimization (Maroušek, 2012a). Therefore, this present study investigated the effect of combined pretreatment of particle size pretreatment technique and nanoparticle additive on biogas and methane yields of *Arachis hypogea* shells that has received little attention. The result of this work is expected to serve as a baseline for further research on the anaerobic digestion process of lignocellulose materials.

## Materials and methods

### Materials

*Arachis hypogea* shells were procured locally and stored in a ventilated area before pretreatment. The inoculum used was prepared from anaerobic co-digestion of kitchen wastes and cow dung. Cow dung was collected from a cattle farm and digested with kitchen waste for 60 days at ambient temperature. Mechanical pretreatment of size reduction was carried out using a hammer mill with screen sizes of 2, 4, 6 and 8 mm.

### Sample analysis

The cellulose, hemicellulose, lignin, total solids, volatile solids, ash content and other elemental composition of the substrate and inoculum were analysed following the Association of Official Analytical Chemist (AOAC) methods (AOAC Official Methods of Analysis, 21^st^ Edition (2019)), and the result is as shown in Table 2.

**Table 2.** Physicochemical composition of the substrate and inoculum.

Parameters	Substrate	Inoculum
Cellulose (%)	38.21	NA
Hemicellulose (%)	18.22	NA
Lignin (%)	27.68	NA
Total solid (%)	95.51	4.01
Volatile solid (%)	91.27	84.83
Ash content (%)	5.96	13.07
Moisture content (%)	4.49	95.99
Nitrogen (%)	1.50	1.48
Carbon (%)	36.39	42.57
Hydrogen (%)	4.79	5.50
Sulphur (%)	0.53	0.60

NA: not applicable.

### Experimental design

The laboratory-batch experiment was performed with a 1-L bio-reactor in batch mode. The effect of combined treatment of particle size and nanoparticle pretreatment on biogas yields of *Arachis hypogea* shells and control was studied. The particle sizes selected for this research were 2, 4, 6 and 8 mm. These were done in modification to the earlier recommendation for the particle selection for anaerobic digestion of lignocellulose materials (Jekayinfa et al., 2020; Menardo et al., 2012; Xiao et al., 2013). The digesters were loaded and labelled as shown in Table 3. 20 mg/L of Fe_3_O_4_ (<50 nm) was added separately to each digester as recommended by Abdelsalam et al. (2016) except for a set that served as a control. The digester performance was measured for the total volume of biogas yield and corrected to the standard pressure (760 mmHg) and temperature (0°C). The setups were replicated twice as recommended by Linke and Schelle (2000).

**Table 3.** Mechanical and nanoparticle pretreatment of the substrate.

Digester	Treatment
A	2 mm particle size + 20 mg of Fe_3_O_4_
B	4 mm particle size + 20 mg of Fe_3_O_4_
C	6 mm particle size + 20 mg of Fe_3_O_4_
D	8 mm particle size + 20 mg of Fe_3_O_4_
E	20 mg Fe_3_O_4_
F	Control

### Experimental setup

A laboratory batch anaerobic digestion process was set up to investigate the influence of pretreatment techniques on biogas and the methane yield of *Arachis hypogea* shells according to Standard Methods VDI 4630 (organischer Stoffe Substratcharakterisierung, 2016). Flasks and round bottom narrow neck bottles of a 1 L inner volume (Schott Duran 10091871, Duran Groups, Hattenbergstrabe, Mainz, Germany) were used as the digester. They were attached to calibrated gas bottles (LMS Boro 3.3, Duran Groups, Hattenbergstrabe, Mainz, Germany) with an inner volume of 0.5 L made from ultra-clear polypropylene graduated cylinder. The gas produced was collected using water displacement methods, and the gas bottles (Schott Duran 10093435, Duran Groups, Hattenbergstrabe, Mainz, Germany) were connected to laboratory bottles of an inner volume of 0.5 L using a silicon pipe. EcoBath thermostatic water batch (30 L) with the control unit software (ECOBATH LABOTECH Model 132A, LABOTECH Ltd, Durban, South Africa) that kept the temperature at mesophilic condition (37 ± 0.02°C) was used to control the digestion temperature. Each digester was fed in a 2:1 ratio of substrate to inoculum determined based on volatile solids content. Fe_3_O_4_ (<50 NM, 544884 – Germany) used for the research was procured from Sigma-Aldrich (pty) Limited, Johannesburg, South Africa. A control sample with inoculum alone was also digested, and the volume of the gas produced was deducted afterward from the substrate sample yield. The volume of biogas released was recorded once daily through the ultra-clear graduated cylinder by considering the volume of water displaced by the gas yield. The composition of the gas yield (CH_4_ and CO_2_) was measured at intervals using BioGas 5000 gas analyzer (Geotech, GA5000, Warwichshire, UK). The experiment was concluded at 35 days of retention time when it was discovered that the volume of biogas released was below 1% of the total biogas yield. The component parts of this setup are readily available in the market, making the design easy and economical.

### Statistical analysis

The focus of the statistical analysis was to determine the influence of the pretreatment methods on biogas and methane yields. Hence, the experiment was replicated twice for statistical analysis purposes, and the result was analysed using Statistical Package for the Social Sciences (SPSS 21.0 version), and the means were sorted out with Duncan Multiple Range Test at a significance level of *p* < 0.05.

## Results

### Effects of pretreatment on biogas and methane production

**Figure 1.**
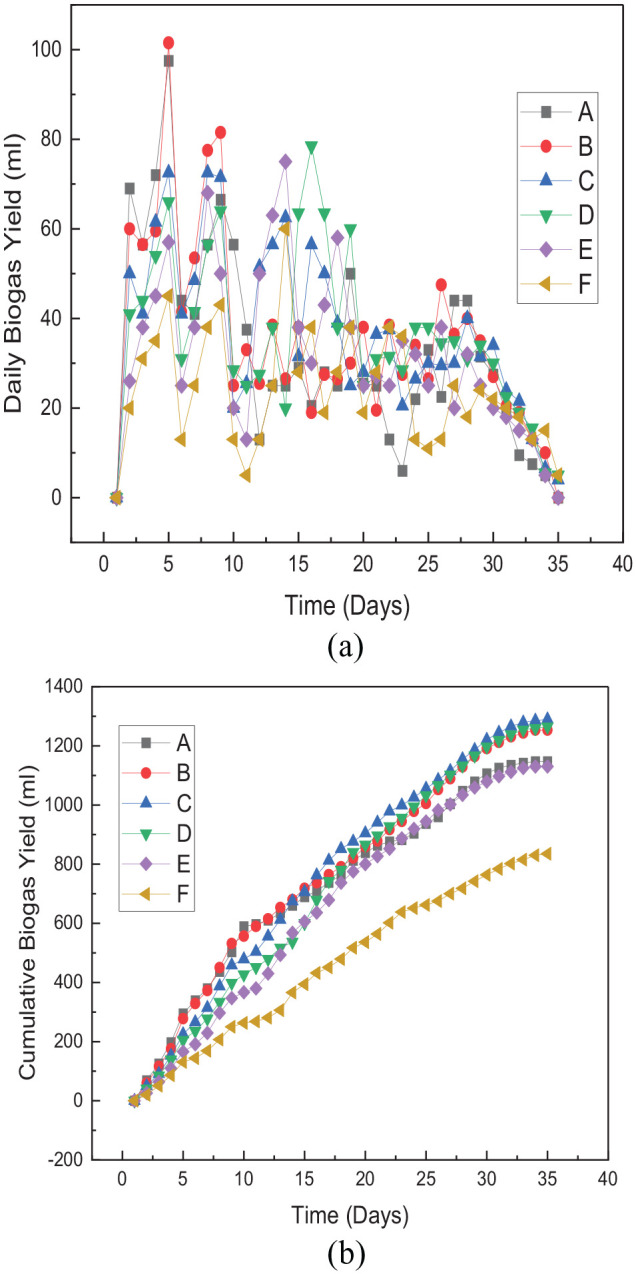
(a) Daily biogas yield from pretreated substrate and control. A: 2 mm particle size + 20 mg Fe_3_O_4_; B: 4 mm particle size + 20 mg Fe_3_O_4_; C: 6 mm particle size + 20 mg Fe_3_O_4_; E: 20 mg Fe_3_O_4_ and F: untreated. (b) Cumulative biogas yield from pretreated substrate and control. A: 2 mm particle size + 20 mg Fe_3_O_4_; B: 4 mm particle size + 20 mg Fe_3_O_4_; C: 6 mm particle size + 20 mg Fe_3_O_4_; E: 20 mg Fe_3_O_4_ and F: untreated.

**Figure 2.**
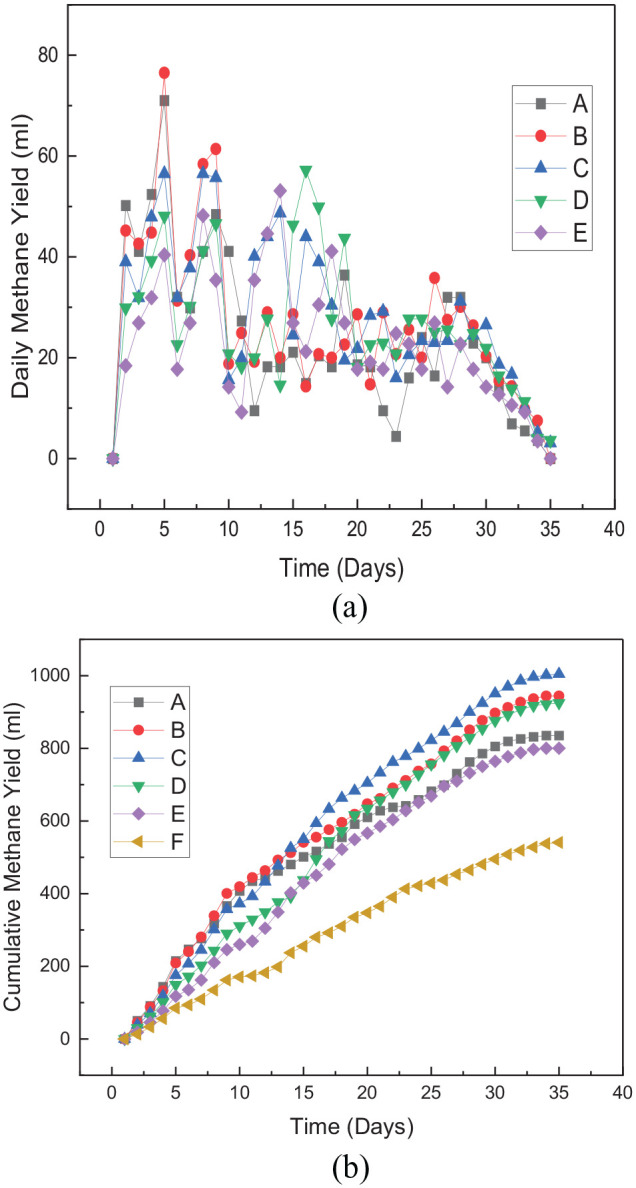
(a) Daily methane yield from pretreated substrate and control. A: 2 mm particle size + 20 mg Fe_3_O_4_; B: 4 mm particle size + 20 mg Fe_3_O_4_; C: 6 mm particle size + 20 mg Fe_3_O_4_; E: 20 mg Fe_3_O_4_ and F: untreated. (b) Cumulative methane yield from pretreated substrate and control. A: 2 mm particle size + 20 mg Fe_3_O_4_; B: 4 mm particle size + 20 mg Fe_3_O_4_; C: 6 mm particle size + 20 mg Fe_3_O_4_; E: 20 mg Fe_3_O_4_ and F: untreated.

The daily and cumulative biogas yields from the pretreated and untreated substrates are illustrated in Figure 2(a) and (b), respectively. All the pretreatments were noticed to enhance the biogas production start-up and lower the retention time of the digestion process. The optimum biogas start-up yield from treatment A was 295 mL biogas on average within the first 5 days of retention time. Treatments B, C, D, E and F produced 277.5, 225, 205, 166 and 86 mL, respectively, within the same retention period. Compared with the control (F), there are 243%, 222.6%, 161.6%, 138.4% and 93% increments for treatments A, B, C, D and E, respectively, in the first 5 days. In addition, the optimum daily biogas yield was recorded from treatment B, which produced about 101.5 mL of biogas from day 5 of the retention period. At the same time, the single pretreatment method (E) yielded an optimum daily yield of 75 mL 9 days later (day 14). At the same time, the optimum daily yield of 60 mL was also recorded on day 14 of the retention period (Figure 1(a)). Moreover, the total biogas yield from all the pretreatments showed that the combination of Fe_3_O_4_ with 6 mm (C) produced the highest biogas yield through 35 days of retention time and was 1289.5 mL biogas compared with other treatments and 54% higher than the control. Cumulatively, treatments A, B, C, D and E were significantly different to each other (*p* < 0.05), produced 1147.5, 1253.5, 1264.5 and 1130 mL, respectively, and they are 37.4%, 50.1%, 51.4% and 35.3% higher than the control (835 mL), respectively (Figure 1(b)). It can be inferred from these results that pretreatment techniques using Fe_3_O_4_ nanoparticles additive improve the cumulative biogas yield from *Arachis hypogea* shells as shown in treatments E and F (*p* < 0.05). Furthermore, it was revealed from the result that combined pretreatment of particle size reduction and Fe_3_O_4_ additive improves the cumulative biogas yield more. All the particle sizes considered in this research showed significant improvement in biogas yield compared with a single pretreatment of Fe_3_O_4_ additive, and 6 mm particle size produced the best yield, followed by 8, 4 and 2 mm in that order.

The optimum daily methane yield was recorded from treatment B (76 mL) at 5 days of retention period. It can be inferred from the results that all the treatment with the combination of particle size reduction and Fe_3_O_4_ released their optimum methane yield on the same hydraulic retention time (day 5), as shown in Figure 2(a). All the pretreatment techniques were noticed to significantly improve the daily methane yield of *Arachis hypogea* shells (*p* < 0.05). The single and combined pretreatment methods were seen to enhance the cumulative methane yield. The cumulative yield recorded for treatments A, B, C, D and E were 835.2, 944.3, 1004.6, 924.9 and 800.3 mL, respectively. These yields were 54.4%, 74.6%, 85.8%, 71.0% and 47.9% increase for treatments A, B, C, D and E, respectively, compared with control with a cumulative yield of 540.8 mL, as illustrated in Figure 2(b). The addition of Fe_3_O_4_ nanoparticles was noticed to increase the cumulative yield of the methane yield (*p* < 0.05) significantly. Combination of particle size reduction with Fe_3_O_4_ nanoparticle addition improved daily and cumulative methane yield. Particle size reduced up to 6 mm, and 20 mg of Fe_3_O_4_ released the highest methane yield. The pretreatment method mentioned above produced the optimum methane yield and was (*p* < 0.05) 835.2, 944.3, 1004.6 and 924.9 mL CH_4_ for A, B, C and D, respectively, in comparison with only Fe_3_O_4_ additive (treatment E). This shows that particle size reduction is crucial to optimizing methane yield from *Arachis hypogea* shells. There is a particular particle size for optimum methane yield during combined pretreatment of particle size reduction and Fe_3_O_4_ nanoparticle addition.

### Methane and carbon dioxide concentration

**Figure 3.**
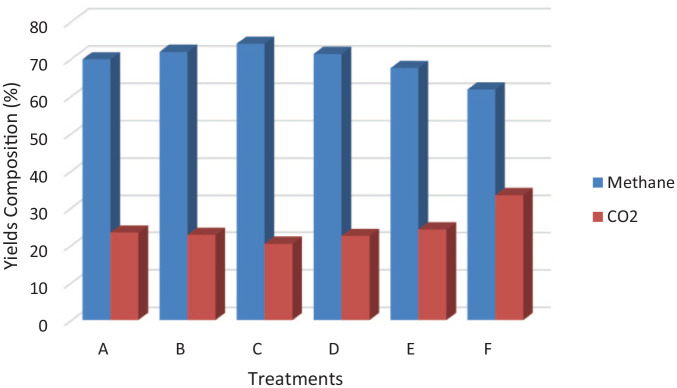
Methane and carbon dioxide concentration from pretreated substrate and control. A: 2 mm particle size + 20 mg Fe_3_O_4;_ B: 4 mm particle size + 20 mg Fe_3_O_4_; C: 6 mm particle size + 20 mg Fe_3_O_4;_ E: 20 mg Fe_3_O_4_ and F: untreated.

The methane and carbon dioxide contents of the single pretreatment, combined pretreatment and control are illustrated in Figure 3. The substrate treated with 6 mm and 20 mg of Fe_3_O_4_ nanoparticles attained the optimum methane yield of 73.95%. There is a significant difference in the percentage of methane yield from treatments A, C, E and F (*p* < 0.05), except for treatments B and D, which have no significant difference (*p* < 0.05). When size reduction with Fe_3_O_4_ nanoparticles was compared with a single treatment of Fe_3_O_4_, it was noticed that a combination of the two pretreatment techniques improved the percentage of methane yield and reduced the carbon dioxide content of the biogas yield. This can be traced to the ability of particle size reduction to make the substrate more accessible for anaerobic digestion microorganisms and the ability of the Fe_3_O_4_ to convert carbon dioxide produced to methane. The optimum methane and lowest carbon dioxide composition were recorded when the particle size was 6 mm. This can be due to the loss of some volatile solid during further size reduction below 6 mm.

Further particle size reduction below 6 mm may also produce inhibitory compounds/materials, hindering methane production. Smaller particle sizes improve the surface area of the substrate and enhance the attachment of the nanoparticles. This can increase hydrolysis, resulting in over-accumulation of volatile fatty acids (VFAs). Over accumulation of VFAs will directly impact the pH of the system, which will, in turn, have a significant effect on the methanogens that produce methane. Anaerobic digestion is most effective when the pH nears the neutral points (6–8) (Ilhan et al., 2017). A low level of the process pH disturbs the methanogenic bacteria growth, which will eventually lower the gas yield and is mainly a result of overloading due to fast hydrolysis of smaller particle sizes (Muvhiiwa et al., 2016).

### The specific biogas and methane yields

**Figure 4.**
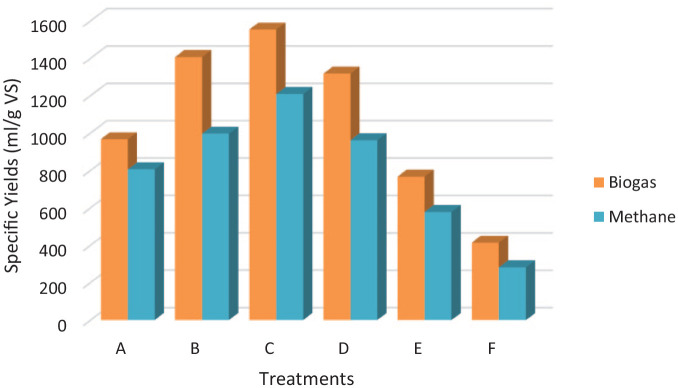
Specific biogas and methane yields from pretreated substrate and control. A: 2 mm particle size + 20 mg Fe_3_O_4_; B: 4 mm particle size + 20 mg Fe_3_O_4_; C: 6 mm particle size + 20 mg Fe_3_O_4_; E: 20 mg Fe_3_O_4_ and F: untreated.

The statistical analysis of the specific biogas and methane yields of the single pretreatment and combined pretreatments confirmed that treatment C released the optimum specific biogas and methane (*p* < 0.05) and were 1552.7 and 1208.5 mL g^−1^ volatile solid, respectively, compared with other treatments as illustrated in Figure 4. The specific biogas and methane yields were significantly different (*p* < 0.05) compared with the control for all the treatments.

### The average biogas and methane yields

The statistical analysis of the average biogas and methane produced showed that the most effective treatment is the combination of Fe_3_O_4_ and 6 mm particle size (treatment C), which produced the average optimum biogas and methane through 35 days of retention time and were 36.8 and 28.7 mL, respectively, as shown in Table 4. The treatment methods were significantly different (*p* < 0.05) compared with single, combined treatments and control. This result indicates that Fe_3_O_4_ has a biostimulating effect on the methanogens, and combining it with a specific particle size made the biostimulating effect more pronounced. The summary of the overall mean of biogas and methane yields as affected by pretreatment methods compared to the control during seven times intervals of the hydraulic retention period is summarized in Table 4.

**Table 4.** Average biogas and methane yields affected by pretreatment methods during different time intervals of HRT.

HRT (days)	Treatments (mL)											
	A		B		C		D		E		F	
	Biogas	CH_4_	Biogas	CH_4_	Biogas	CH_4_	Biogas	CH_4_	Biogas	CH_4_	Biogas	CH_4_
1–5	295	214.7	277.5	209.1	225	175.3	205	149.4	166	117.6	131	85
6–10	559.5	407.2	556.5	419.5	478.5	372.8	426.5	310.8	367	260	263	170.5
11–15	689	501.5	718.0	541	706	550	600.5	437.6	606	429.2	394	255.3
16–20	838	610	859.0	647.2	904.5	704.7	865.5	634.2	800	566.6	536	347.2
21–25	937	682.1	1005.0	757.2	1055.5	822.3	1032.5	755.9	944	668.6	662	428.7
26–30	1106.5	805.4	1191.0	897.3	1220.5	950.9	1197	875.8	1079	764.3	764	494.8
31–35	1147.5	835.2	1253.5	944.3	1289.5	1004.6	1264.5	924.9	1130	800.3	835	540.8
Average	32.8	23.9	35.8	27.0	36.8	28.7	36.1	26.4	32.3	22.9	23.9	15.5

A: 2 mm + 20 mg Fe_3_O_4_; B: 4 mm + 20 mg Fe_3_O_4_; C: 6 mm + 20 mg Fe_3_O_4_; D: 8 mm + 20 mg Fe_3_O_4_; E: 20 mg Fe_3_O_4_; F: control and HRT: Hydraulic Retention Time.

## Discussion

This study has confirmed that combined pretreatment of particle size reduction and Fe_3_O_4_ nanoparticle produced the optimum biogas and methane yields compared to single treatment of Fe_3_O_4_ and control, where the statistical analysis indicates a significant difference (*p* < 0.05) with all the treatments. This is in line with Zaidi et al. (2019), who recorded that combining microwave pretreatment with Fe_3_O_4_ nanoparticles released the optimum biogas and methane yields compared with the individual ones and control. In similar research, Fe_3_O_4_ was also combined with ultrasonic, microwave and ozone pretreatment during the anaerobic digestion of macroalgae. The results show an improvement in biogas and methane yields from combined pretreatment compared to individual treatment (Nemr et al., 2021), which corroborates this work’s findings. Abdelsalam et al. (2016) reported 73% biogas and 115.66% methane yield when 20 mg/L of Fe_3_O_4_ was added to cattle dung slurry. Methane yield was increased by 117% when 100 mg/g total suspended solid (TSS) was added to waste-activated sludge in the same experimental condition with this work. The percentage of biogas and methane yields from this research was lesser than these improvements at Fe_3_O_4_ alone and combined with particle size reduction. This result negates what was recorded by Men et al. (2020) that adding zero-valent iron during anaerobic digestion improved the anaerobic digestion of easily digestible substrates but inhibited the digestion of lignocellulose materials. But, Olatunji et al. (2021) have established the effect of nanomaterials on biogas and methane yields. The influence of nanoparticles depends on the particle size and concentration of the nanomaterials and the structure of the substrate. A similar result was recorded by Ossinga when an iron oxide nanoparticle was used to pretreat winery solid and sorghum stover (Ossinga, 2020). Biogas was increased by 1.27 times when Fe_3_O_4_ was added to the anaerobic digestion of sludge (Suanon et al., 2017). It was noticed by Casals et al. (2014) that Fe^2+^ behaves as a unique source that breaks down the organic materials and improves biogas and methane yields in the anaerobic digester.

An earlier report by Wang et al. (2016) showed that Fe_3_O_4_ nanoparticles improve the reaction kinetics, enhance yields and reduce the retention period. This result also corroborates what was earlier recorded that the Fe_3_O_4_ additive as trace metal could lower the retention period of mixed culture (Krongthamchat et al., 2006). This research indicated an optimum methane yield with 20 mg/L Fe_3_O_4_ magnetic nanoparticles improved by 85.8%, which is lesser than what was reported by Abdelsalam et al. (2016) when the 20 mg/L Fe_3_O_4_ was added to cattle dung slurry. This difference may be connected to the lignocellulosic nature of *Arachis hypogea* shells. But the percentage increase recorded in this research is higher than what was reported by Feng et al. (2014), who reported a 43.5% increase when 20 g/L of Fe_3_O_4_ was introduced to waste-activated sludge. These results showed that Fe_3_O_4_ magnetic nanoparticles at a dose of 20 mg/L could improve anaerobic digestion and produce higher biogas and methane and organic matter degradation. The performance improvement recorded can be traced to the availability of Fe^+2^/Fe^+3^ ions, added into the digester in nanoparticle form and serve as the growth enhancement element for the anaerobic digestion microorganisms (Abdelsalam et al., 2016). In addition, Fe can also serve as an electron donor for lowering the carbon dioxide into methane via autotrophic methanogenesis, thereby increasing the methane percentage following Equations (1) and (2) as reported by Feng et al. (2014):

(1) $${\mathrm{CO}}_{2}+4{\mathrm{Fe}}^{0}+8H^{+}={\mathrm{CH}}_{4}+4{\mathrm{Fe}}^{2+}+2H_{2}O$$

(2) $${\mathrm{CO}}_{2}+4H_{2}={\mathrm{CH}}_{4}+2H_{2}O$$

It is evident from this work that the Fe_3_O_4_ treatment only (treatment E) releases the lowest biogas and methane yields after the control. The source of carbon during the digestion is the *Arachis hypogea* shells, which were not easy to degrade due to their complex lignocellulose nature. For easy accessibility of anaerobic digestion microorganisms, there is a need to increase the available surface area of the substrate for cellulose availability, enhance hydrolysis and enhance the deposition of solid iron particulates on the cell surface of the substrate. It can be deduced from this research that particle sizes influence the biogas and methane yields, as reported earlier (Maroušek, 2012b; Olatunji et al., 2022a). This work showed that 6 mm particle size (treatment C) produced the best biogas and methane yields. This result agreed with what was recorded by Motte et al. (2015), who observed that the highest biogas and methane yields were attained at particle size that is not close to fine form. Another similar research reported that the biogas and methane yields were enhanced until the particle size was 6 mm, but below 6 mm particle size, the yield started to reduce (Herrmann et al., 2012). Jekayinfa et al. (2020) reported 6 mm particle size as the optimum value for fresh biogas and methane yields of lignocellulose material. This result also supports what was reported by an earlier researcher when particle size was considered (Olatunji et al., 2022b). But this result contradicts what was recorded by Menardo et al. (2012) that a particle size of 0.5 cm produced the optimum biogas and methane yields.

Similarly, it was also reported that a particle size of between 1 and 2 mm is the most effective particle size for the digestion of lignocellulose feedstock (Montgomery and Bochmann, 2014). Several other researchers have noticed that smaller particle sizes produce better biogas and methane yields than their bigger sizes (Kirby et al., 2020; Kulichkova et al., 2020; Prade et al., 2019). It was noticed that mechanical pretreatment enhances the biogas and methane yields of lignocellulose materials like wheat straw and barley. In contrast, it does not for lignocellulose materials like rice straw and maize stalk (Menardo et al., 2012). In line with this assertion, groundnut shell has been established as a lignocellulose material that particle size reduction beyond a particular size does not favour its biogas and methane yields (Jekayinfa et al., 2020). This agrees with what was earlier noticed: if the substrate can be reduced to a point at which it is easy to degrade, overloading of digesters is possible with high organic loading, especially in the batch system. This may cause an imbalance between the acidogenesis/acetogenesis and methanogenesis stage, leading to the substantial accumulation of VFAs, reduced the alkalinity and pH values, and consequently inhibiting the methanogenesis stage (Braz et al., 2019). Another possibility of having a better result before the smaller particle sizes can be due to the production of the inhibitory compound during further size reduction. Smaller particles improve the surface area of the substrate and enhance the nanoparticle attachment. This enhances the hydrolysis rate, leading to over-accumulation of VFAs. Over accumulation of VFAs will affect the pH of the process, which will, in turn, have a negative influence on the methanogens that release biogas. The anaerobic digestion process is most efficient when the pH of the process is closer to neutral points (6–8) (Ilhan et al., 2017). Lower or higher pH of the process due to VFAs accumulation will affect the methanogenic bacteria development, which will eventually lower gas yield. The lower gas yield below 6 mm particle sizes is mainly a result of overloading due to fast hydrolysis of the smaller particle sizes (Muvhiiwa et al., 2016). Microbial community analysis has shown that the addition of Fe_3_O_4_ drastically changed the bacterial population in the methanogenic acetate-degrading cultures (Yamada et al., 2015), and syntropic microorganisms can attach to surfaces of relatively larger (mm scale) organic materials. Therefore the addition of Fe_3_O_4_ to the 6 mm particle size improves the DIET better than other particle sizes (Liu et al., 2015). This can be investigated further in the subsequent research. Economically, having the best yields at 6 mm particle size could be an advantage as the energy required for further size reduction will be saved, thereby reducing the cost of energy which will, in turn, make the process more economical. Fe_3_O_4_ is not encouraged when the digestate is expected to be added to agricultural land to serve as a nutrient source for the plants. It has been reported that traces of iron from the FeO(OH) changed the phosphorous in the soil into iron phosphates (FeP), which makes phosphorous unavailable to agricultural plants (Maroušek et al., 2020).

The technology applied in this work does not require expensive construction nor other additional energy, catalysts or chemicals and is similar to what was used in earlier literature (Maroušek, 2012b). However, particle size reduction needs some cost-effective energy at a laboratory scale. Economically, the technique requires further investigation at a commercial scale before it can be recommended. The detailed parameters related to particle sizes and Fe_3_O_4_ additives show the influence of these operating parameters on the yield, as reported earlier (Abdelsalam et al., 2016; Menardo et al., 2012; Olatunji et al., 2022b). The results showed that combined particle size reduction with Fe_3_O_4_ influences the biogas and methane yields more than the single pretreatment of Fe_3_O_4_ nanoparticle additive. Compared with other pretreatment techniques and lignocellulose materials, the biogas and methane yields are significantly higher (Ossinga, 2020; Siddhu et al., 2016; Xu et al., 2019).

Nevertheless, it must be noticed that the feedstock investigated in this work is *Arachis hypogea* shells, and the results recorded may not be the same when another lignocellulose substrate is considered. Another limitation of this work is that the removal efficiencies of lignin, hemicellulose and cellulose were not considered after the anaerobic digestion process. This needs to be considered in future works.

## Conclusion

In this study, Fe_3_O_4_ nanoparticle single pretreatment of *Arachis hypogea* shells improved the biogas yields, and its combination with particle size reduction also showed improved yields. This research indicated that particle size reduction pretreatment is required before Fe_3_O_4_ nanoparticle additive. It can be concluded from this research that the combination of 6 mm particle size of *Arachis hypogea* shells with 20 mg/L Fe_3_O_4_ nanoparticle additive produced the optimum biogas and methane yields. This study can be applied to some lignocellulose materials with high resistant cell walls or cellulose structures to improve the hydrolysis stage and total energy recovery.
